# Association between milk consumption and kidney stones in U.S. adults: results from NHANES 2007–2018

**DOI:** 10.3389/fnut.2024.1394618

**Published:** 2024-05-15

**Authors:** Zhouzhou Xie, Yiming Zhuang, Shansen Peng, Xiaoqi Zhou, Guihao Zhang, Huiming Jiang, Changyi Zhang, Nanhui Chen

**Affiliations:** ^1^Meizhou Clinical Institute of Shantou University Medical College, Meizhou, China; ^2^Department of Urology, Meizhou People's Hospital (Meizhou Academy of Medical Sciences), Meizhou, China; ^3^Department of Cardiology, Second Affiliated Hospital of Shantou University Medical College, Shantou, China

**Keywords:** milk consumption, kidney stones, NHANES, cross-sectional study, female

## Abstract

**Background:**

Dietary strategies play a crucial role in the prevention of kidney stones. While milk is known for its rich nutritional content, its impact on kidney stone formation remains unclear. This study aimed to examine the relationship between milk consumption and the risk of kidney stones among U.S. adults.

**Methods:**

We included 24,620 participants aged 20 and older from the National Health and Nutrition Examination Survey (2007–2018). Milk consumption was defined based on each participant’s response to the questionnaire item on “Past 30 day milk product consumption.” Kidney stones history was self-reported by participants. The analysis employed weighted multivariate logistic regression models, followed by subgroup analyses for result validation, and explored the age-related dynamics of milk consumption’s effect on kidney stone risk using a restricted cubic spline model.

**Results:**

Adjusted findings revealed that higher milk intake was associated with a decreased risk of kidney stones (odds ratio [OR] = 0.90, 95% confidence interval [CI] 0.85–0.96), notably among women (OR = 0.86, 95% CI 0.80–0.92) but not significantly in men (OR = 0.94, 95% CI 0.86–1.02). Smoothed curves across all ages showed that women consuming milk had a lower incidence of kidney stones than those who did not, particularly with regular consumption.

**Conclusion:**

This study uncovered that across all age groups, higher frequency of milk consumption in women is associated with a reduced risk of kidney stones. However, further prospective cohort studies are needed to confirm this finding.

## 1 Introduction

Kidney stones is a prevalent condition within the urinary tract system, characteristically presenting with symptoms such as unilateral abdominal discomfort, infections of the urinary tract, and hematuria. In its severest form, it can precipitate renal failure, significantly impairing patient quality of life (1, 2). Epidemiological data from recent decades indicate a notable surge in both the incidence and prevalence of kidney stones, with global incidence rates estimated between 1 and 15%. Specifically, the incidence in the United States is reported at 11.0% (3, 4). The therapeutic landscape for kidney stones predominantly involves costly surgical interventions. In the United States, the fiscal implications of kidney stones are profound, with annual healthcare costs attributed to this condition reaching into the billions and projected to escalate further, thereby imposing a considerable economic burden on both societal and individual levels (5). This scenario accentuates the necessity for more rigorous development and implementation of preventative strategies against kidney stones.

The pathogenesis of kidney stones is multifactorial, with genetic, dietary, behavioral, and inflammatory factors all playing contributory roles over time. Hence, targeting these underlying etiological factors or risk elements is recognized as the most effective method for prevention (6). Dietary interventions are highlighted as a critical preventative measure against the onset of kidney stones in daily life (7). Milk, an essential component of the human diet, is a rich source of proteins, lipids, a variety of vitamins, calcium, magnesium, and other trace elements (6). Research to date has demonstrated milk’s potential in mitigating the risk of various chronic conditions, including cardiovascular and cerebrovascular diseases, dementia, diabetes, and cancer (8–12). However, the impact of milk consumption on the risk of developing kidney stones remains unexplored. Disturbances in calcium and vitamin D metabolism within the human body are closely associated with the incidence of kidney stones (13). In the past, high dietary calcium intake was frequently perceived as a risk factor for the development of kidney stones. Contrarily, an increasing body of research collectively suggests that a high daily intake of dietary calcium is beneficial for the prevention of kidney stones (14–18). Additionally, preliminary studies suggest that increasing the intake of fluids, dietary magnesium may reduce the risk of kidney stones (6, 19). Moreover, the Dietary Approaches to Stop Hypertension diet is associated with a decreased risk of kidney stones, with low-fat dairy products being a part of this dietary approach (20). Given milk’s established profile as a healthful beverage, this generates a scientific hypothesis that augmented milk consumption may confer protective effects against the formation of kidney stones.

Considering these considerations, an investigation into the relationship between milk intake and the risk of kidney stones could provide critical insights for the formulation of preventive measures, thereby fostering the advancement of global public health initiatives. This research employs a cross-sectional design, leveraging data from the National Health and Nutrition Examination Survey (NHANES) covering the years 2007 to 2018, to examine this potential correlation.

## 2 Materials and methods

### 2.1 Study participants

NHANES, conducted by the National Center for Health Statistics in the United States, is a continuous, stratified, multistage sampling survey that integrates structured interviews and physical examinations to comprehensively assess the health and nutritional status of the American adult and child populations. The sampling strategy and detailed information used in this study were obtained from the NHANES website.^1^ Written consent was obtained from all participants involved in NHANES.

**Figure 1:**
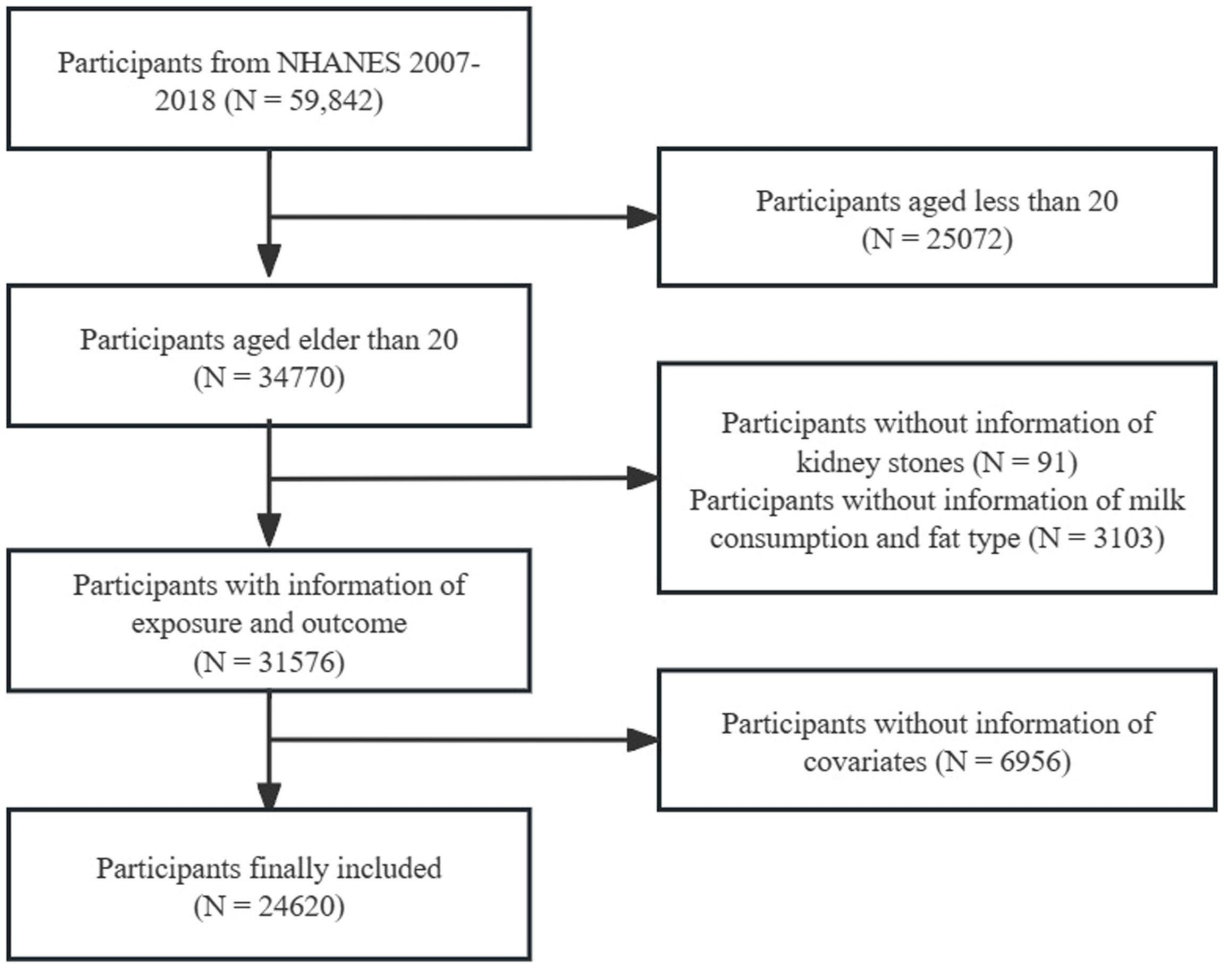
Flow chart for inclusion and exclusion of study participants. NHANES, National Health and Nutrition Examination Survey.

In this cross-sectional study, we utilized data from six consecutive cycles of NHANES spanning from 2007 to 2018. During these 12 years, a total of 59,842 participants were included. Initially, 25,072 participants under the age of 20 were excluded. Subsequently, we excluded 91 participants with incomplete kidney stones history information and 3,103 participants with incomplete data on milk consumption (consumption frequency and milk fat content), as well as 6,956 participants lacking information on covariates. Ultimately, this study identified 24,620 participants who met the inclusion criteria (Figure 1).

### 2.2 Exposure and outcome definitions

As the exposure variable, milk consumption was defined based on participants’ responses to the questionnaire item “Past 30 day milk product consumption.” Based on their responses, participants were categorized into four groups: (1) never, (2) rarely: less than once a week, (3) sometimes: once a week or more, but less than once a day, and (4) often: once a day or more. Additionally, we classified the type of milk consumed by participants into three categories based on the fat content of the milk: “1% fat milk” or “2% fat milk” was defined as semi-skimmed milk, while the other two categories were whole milk and skim milk.

Kidney stones history was designed as the outcome variable, determined by participants’ responses to the survey question “Ever had kidney stones?” The reliability of self-reported kidney stones history has been validated by relevant studies (21).

### 2.3 Covariates definition

To account for potential confounding factors, this study incorporated the following covariates based on insights from other research (22): age, body mass index (BMI), gender, race (non-Hispanic Black, non-Hispanic White, Mexican American, and other), marital status (married/living with partner and living alone), educational level (less than high school, high school or equivalent, and college degree or higher), household income and Poverty Income Ratio (PIR), history of smoking, history of alcohol consumption, recreational activities (inactive or moderate and vigorous), hypertension, and diabetes history. The PIR was divided into three groups: low (≤ 1.30), middle (>1.30, ≤ 3.50), and high (> 3.5). History of smoking, alcohol consumption, and hypertension were all recorded as “Yes/No,” while diabetes history was recorded as “Yes/No/Borderline.”

### 2.4 Statistical analysis

To enhance the national representativeness of our sample, we employed weighted analysis to mitigate the effects of the complex multi-stage sampling design, adhering to the sample weighting data, and merging methods recommended by NHANES. In the baseline characteristics table, continuous variables were presented using a weighted mean with standard error (SE), whereas categorical variables were reported as the number of observations with weighted percentages (%). Weighted linear regression for continuous variables and weighted Chi-square tests for categorical variables were utilized to assess differences between populations with and without kidney stones formation.

Considering the sample weights in the survey, we employed weighted multivariate logistic regression models to explore the relationship between milk consumption and the prevalence of kidney stones. We utilized three models in our analysis: Model 1 did not adjust for any covariates, Model 2 was adjusted for age, gender, and race only, and Model 3 was further adjusted for a comprehensive set of variables including age, gender, race, educational level, marital status, PIR, BMI, smoking history, alcohol consumption history, physical activity (leisure activities), hypertension, and diabetes. Based on the results of the weighted logistic regression analysis, we also conducted trend tests to better assess the overall impact brought by changes in milk consumption.

To assess the robustness of the relationship between milk consumption and the prevalence of kidney stones across different populations, subgroup analyses were conducted within the context of regression Model 3, utilizing interaction terms to identify specific population segments where the relationship might differ. Each subgroup included an interaction term, and the interaction among subgroups was evaluated using the log-likelihood ratio test. In these subgroup analyses, continuous variables were treated as categorical, with age being divided into two groups at the threshold of 60 years and BMI at the threshold of 25 kg/m^2. Regression Model III was then reapplied to study the specific impact of milk consumption on the prevalence of kidney stones in these populations. Restricted cubic splines (RCS) model is a powerful tool for describing the dose–response relationships between continuous exposure and outcomes (23). We aim to use this model to explore the effects of certain continuous variables on the incidence of kidney stones across different levels of milk consumption. Recently, a study by Park et al., which conducted a dose–response analysis on the impact of varying levels of coffee consumption across all ages on mortality, clearly demonstrated the effect on mortality rates (24). Given that age has been identified as a significant factor in the incidence of kidney stones (21), RCS model was utilized to plot smooth curves to better explore the impact of milk consumption on the prevalence of kidney stones across different ages. Following this, based on the curves, piecewise linear regression models were applied to identify the threshold effect of age on the prevalence of kidney stones and to detect the inflection point where the relationship begins to change significantly (25).

All statistical analyses in this study were conducted using R software^2^ (version 4.3.2) and EmpowerStats^3^ (X&Y Solutions, Inc., Boston, MA) for both statistical analysis and graphing purposes. Statistical significance was determined by a two-sided p-value of 0.05.

## 3 Results

### 3.1 Characteristics of the study population

The study included a total of 24,620 participants, among which 2,396 individuals reported having been diagnosed with kidney stones (Table 1). Statistically significant differences were observed across most baseline characteristics when participants were divided into groups based on their history of kidney stones. Compared to the non-kidney stones group, kidney stones patients were older on average (53.27 years), had a higher average BMI (30.83 kg/m^2), a higher proportion of males (55.92%), a higher proportion of non-Hispanic whites (78.55%), a higher proportion of those who were not single (69.75%), a higher proportion of smokers (50.76%), a higher proportion of individuals engaging in less vigorous activity (81.16%), and higher proportions of individuals with hypertension (47.37%) and diabetes (18.33%). In this study, the focus was particularly on the association between milk consumption and the prevalence of kidney stones. The data showed that individuals in the kidney stones group consumed milk less frequently across all categories (Rarely = 15.54%, Sometimes = 28.06%, Often = 33.58%), and a higher proportion of individuals never consumed milk (Never = 22.82%) compared to the non-kidney stones group (Never = 18.85%).

**Table 1:** Baseline characteristics of participants between 2007 and 2018 (n = 24,620).

**Characteristic**	**None-stone formers**	**Stone formers**
	**(*N* = 22,224)**	**(*N* = 2,396)**
Age (years), mean (SE)***	46.87 (0.50)	53.27 (0.71)
BMI(kg/m^2^), mean (SE)***	29.06 (0.17)	30.83 (0.35)
Gender, *n* (%)***		
Male	10,919 (48.71)	1,365 (55.92)
Female	11,305 (51.29)	1,031 (44.08)
Race, *n* (%)***		
Mexican American	5,395 (13.53)	549 (10.35)
Non-Hispanic White	9,521 (68.07)	1,373 (78.55)
Non-Hispanic Black	4,744 (10.89)	291 (5.33)
Other	2,564 (7.52)	183 (5.77)
Marital status, *n* (%)***		
Married/with partner	13,171 (63.01)	1,540 (69.75)
Single	9,053 (36.99)	856 (30.25)
Education level, *n* (%)		
Less than high school	5,229 (15.13)	579 (15.06)
High school or equivalent	5,207 (23.53)	548 (23.31)
College or above	11,788 (61.34)	1,269 (61.64)
PIR, *n* (%)		
<= 1.30	7,202 (21.82)	745 (19.39)
> 1.30, < = 3.50	8,345 (35.23)	933 (37.41)
> 3.50	6,677 (42.95)	718 (43.21)
Smoking, *n* (%)***		
No	12,257 (55.50)	1,132 (49.24)
Yes	9,967 (44.50)	1,264 (50.76)
Drinking, *n* (%)		
No	5,550 (19.62)	590 (19.80)
Yes	16,674 (80.38)	1806 (80.20)
Activity, *n* (%)***		
Inactive or moderate	17,331 (73.70)	2041 (81.16)
Vigorous	4,893 (26.30)	355 (18.84)
Hypertension, *n* (%)***		
No	14,417 (69.24)	1,177 (52.63)
Yes	7,807 (30.76)	1,219 (47.37)
Diabetes, *n* (%)***		
No	19,032 (89.13)	1765 (78.20)
Yes	2,703 (8.86)	550 (18.33)
Borderline	489 (2.01)	81 (3.47)
Milk consumption, *n* (%)**		
Never	4,378 (18.85)	517 (22.82)
Rarely	3,802 (17.36)	383 (15.54)
Sometimes	6,329(28.41)	636 (28.06)
Often	7,715 (35.39)	860 (33.58)

BMI, body mass index; PIR, poverty income ratio; SE, standard error; N/n, number of observed; %, weighted percentage; Rarely, less than once a week; Sometimes: once a week or more, but less than once a day; Often, once a day or more. *p < 0.05; **p < 0.01; ***p < 0.001. For continuous variables: weighted mean with standard error, P was by weighted linear regression. For categorical variables: number of observed with weighted percentage, P was by weighted Chi-square test.

### 3.2 Associations between milk consumption and kidney stones

Weighted multivariable logistic regression analyses demonstrate a notable inverse relationship between the frequency of milk consumption and the risk of kidney stones (Table 2). This association remains significant across several models: an unadjusted Model 1 [odds ratio (OR) = 0.94, 95% confidence interval (CI) 0.89–0.99], a minimally adjusted Model 2 (OR = 0.91, 95% CI 0.86–0.96), and a fully adjusted Model 3 (OR = 0.90, 95% CI 0.85–0.96). Compared to individuals who never consume milk, those who frequently consume milk (‘Often’) exhibit a significantly lower risk of developing kidney stones (OR = 0.71, 95% CI 0.60–0.83).

Moreover, the variety of milk types, specifically whole milk, semi-skimmed milk, and skim milk, which are commonly consumed in daily life, prompted a stratified analysis based on the fat content of milk. Overall, in Model 3, regardless of the type of milk, the frequency of milk consumption was inversely associated with the prevalence of kidney stones. Intriguingly, the protective effect of milk against kidney stones appears to strengthen with increasing degrees of fat removal. The results showed that whole milk (OR = 0.93, 95% CI 0.88–0.99), semi-skimmed milk (OR = 0.91, 95% CI 0.86–0.97), and skimmed milk (OR = 0.80. 95% CI 0.72–0.89).

### 3.3 Subgroup analysis

**Figure 2:**
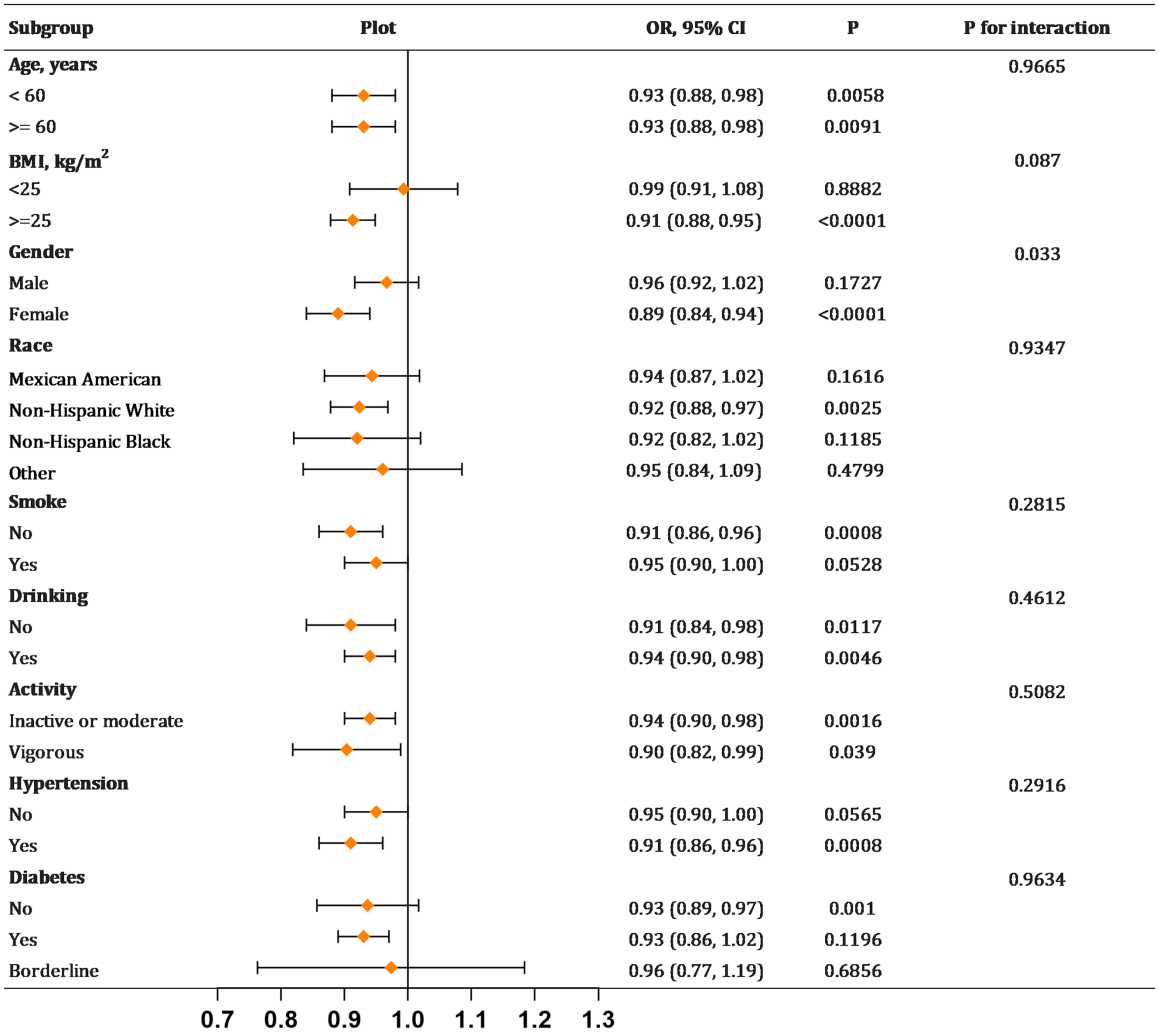
Subgroup analyses for the relationship between milk consumption and kidney stones. BMI, body mass index. Each stratification adjusted for all the factors (age, gender, race, education level, marital status, PIR, BMI, smoking, drinking, activity, hypertension, diabetes) except the stratification factor itself in model.

Subsequent subgroup analyses were conducted to evaluate the robustness of the link between milk intake and the incidence of kidney stones across diverse demographic cohorts (Figure 2). Beyond the stratification variables themselves, adjustments were made for a comprehensive set of covariates including age, sex, race, educational attainment, marital status, PIR, BMI, smoking status, alcohol use, physical activity, hypertension, and diabetes mellitus. These analyses revealed that the inverse relationship between milk consumption and kidney stone prevalence remained unaltered across most subgroups, with no significant interaction effects observed (*P* for interaction >0.05). However, gender emerged as a significant modifier of the relationship between milk intake and kidney stone risk, denoting a specific interaction (*P* for interaction = 0.033). Next, our investigation will specifically explore the differential impact of milk consumption on kidney stone formation across genders.

**Table 2:** Weighted multivariable logistic regression for the association between milk consumption and kidney stones.

**Milk consumption**	**Participants, *N***	**Stone formers, %**	**OR (95% CI)**		
			Model 1	Model 2	Model 3
**Total**					
Never	4,895	12.1	1.00 (reference)	1.00 (reference)	1.00 (reference)
Rarely	4,185	9.24	0.74 (0.62, 0.88)	0.79 (0.66, 0.94)	0.75 (0.63, 0.89)
Sometimes	6,965	10.09	0.82 (0.69, 0.97)	0.82 (0.68, 0.98)	0.79 (0.66, 0.94)
Often	8,575	9.74	0.78 (0.67, 0.92)	0.71 (0.61, 0.84)	0.71 (0.60, 0.83)
*P* for trend	–	–	0.0302	0.0007	0.0009
Increase per level	–	–	0.94 (0.89, 0.99)	0.91 (0.86, 0.96)	0.90 (0.85, 0.96)
**Whole milk**					
Never	4,895	12.1	1.00 (reference)	1.00 (reference)	1.00 (reference)
Rarely	1,530	9.8	0.79 (0.59, 1.06)	0.93 (0.69, 1.25)	0.92 (0.68, 1.24)
Sometimes	2,322	9.41	0.75 (0.60, 0.95)	0.84 (0.67, 1.06)	0.87 (0.69, 1.09)
Often	2,516	9.55	0.77 (0.62, 0.95)	0.78 (0.62, 0.97)	0.82 (0.65, 1.02)
*P* for trend	–	–	0.0017	0.0075	0.0314
Increase per level	–	–	0.90 (0.85, 0.96)	0.92 (0.87, 0.98)	0.93 (0.88, 0.99)
**Semi-skimmed milk**					
Never	4,895	12.1	1.00 (reference)	1.00 (reference)	1.00 (reference)
Rarely	2,308	9.1	0.73 (0.58, 0.91)	0.75 (0.60, 0.93)	0.69 (0.56, 0.86)
Sometimes	3,876	10.68	0.87 (0.71, 1.07)	0.85 (0.69, 1.05)	0.80 (0.64, 0.99)
Often	4,755	10.47	0.85 (0.71, 1.02)	0.76 (0.63, 0.92)	0.74 (0.61, 0.89)
*P* for trend	–	–	0.1598	0.0135	0.0064
Increase per level	–	–	0.96 (0.90, 1.02)	0.92 (0.87, 0.98)	0.91 (0.86, 0.97)
**Skim milk**					
Never	4,895	12.1	1.00 (reference)	1.00 (reference)	1.00 (reference)
Rarely	347	8.39	0.67 (0.42, 1.05)	0.66 (0.41, 1.05)	0.67 (0.41, 1.09)
Sometimes	767	9.08	0.73 (0.50, 1.05)	0.65 (0.45, 0.94)	0.64 (0.44, 0.95)
Often	1,304	7.91	0.62 (0.48, 0.81)	0.51 (0.38, 0.67)	0.52 (0.38, 0.70)
*P* for trend	–	–	0.0009	<0.0001	0.0001
Increase per level	–	–	0.85 (0.78, 0.93)	0.80 (0.72, 0.88)	0.80 (0.72, 0.89)

N, number of observed; %, weighted percentage; OR, odds ratio; 95%CI, 95% confidence interval; Rarely, less than once a week; Sometimes, once a week or more, but less than once a day; Often, once a day or more. Model 1: no covariates were adjusted. Model 2: age, gender, and race were adjusted. Model 3: age, gender, race, educational level, marital status, BMI, PIR, smoking, drinking, activity, hypertension, and diabetes were adjusted.

### 3.4 Association between milk consumption and kidney stones based on gender

In the regression model (Table 3), there appears to be no significant association between the frequency of milk consumption and the prevalence of kidney stones among the male population (P for trend = 0.1647), with a notable negative correlation observed only within the ‘Rarely’ category of milk consumption (OR = 0.71, 95%CI 0.51–0.99). Conversely, for the female cohort, milk consumption significantly reduces the risk of kidney stone formation (OR = 0.86, 95%CI 0.80–0.92). Importantly, this trend becomes particularly evident with increasing frequency levels: ‘Rarely’ (OR = 0.77, 95%CI 0.60–0.99), ‘Sometimes’ (OR = 0.71, 95%CI 0.57–0.89), ‘Often’ (OR = 0.62, 95%CI 0.49–0.77).

**Table 3:** Weighted multivariable logistic regression for the association between milk consumption and kidney stones based on gender.

**Milk consumption**	**Male (*N* = 12,284)**			**Female (N = 12,336)**		
	**Participants, *N***	**Stone formers, %**	**OR (95% CI)**	**Participants, *N***	**Stone formers, %**	**OR (95% CI)**
Never	2,339	13.2	1.00 (reference)	2,556	11.13	1.00 (reference)
Rarely	1981	9.29	0.71 (0.51, 0.99)	2,204	9.19	0.77 (0.60, 0.99)
Sometimes	3,511	11.69	0.85 (0.64, 1.14)	3,454	8.45	0.71 (0.57, 0.89)
Often	4,453	11.59	0.77 (0.59, 1.01)	4,122	7.77	0.62 (0.49, 0.77)
*P* for trend	–	–	0.1647	–	–	0.0001
Increase per level	–	–	0.94 (0.86, 1.02)	–	–	0.86 (0.80, 0.92)

N, number of observed; %, weighted percentage; OR, odds ratio; 95%CI, 95% confidence interval; Rarely, less than once a week; Sometimes, once a week or more, but less than once a day; Often, once a day or more. Age, race, educational level, marital status, BMI, PIR, smoking, drinking, activity, hypertension, and diabetes were adjusted in model.

### 3.5 With the age changing, association between milk consumption and kidney stones based on gender

**Figure 3:**
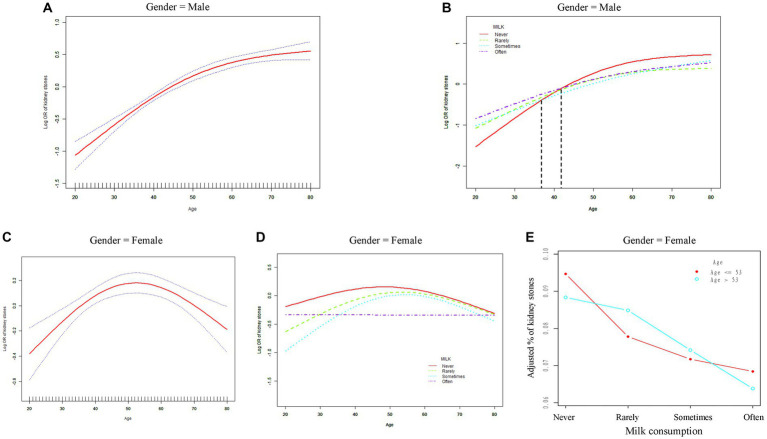
The relationship between age and the risk of kidney stones based on gender. **(A,B)** Smooth fitting curves in male. **(C-E)** Smooth fitting curves in female. OR, odds ratio; Rarely, less than once a week; Sometimes, once a week or more, but less than once a day; Often, once a day or more. Race, educational level, marital status, BMI, PIR, smoking, drinking, activity, hypertension, and diabetes were adjusted in model.

As it is widely recognized, age is closely associated with the occurrence of kidney stones (21). Thus, to investigate the age-related variations in the impact of milk consumption on the prevalence of kidney stones, we employed RCS model to generate smooth fitting curves. The results (Figures 3A–D) demonstrated distinct gender-specific patterns in the influence of age on kidney stone prevalence. In males, the prevalence of kidney stones gradually increased with advancing age (Figure 3A). This trend remained consistent across different frequencies of milk consumption groups; however, milk consumption did not consistently reduce the prevalence of kidney stones in males across all age groups. Notably, we observed that milk consumption appeared to have a promotive effect on kidney stone occurrence in males until approximately the age of 40, after which this phenomenon reversed (Figure 3B).

Moreover, we identified a nonlinear relationship between age and kidney stone prevalence in females, characterized by a clear inflection point in the curve (Figure 3C). Subsequently, through threshold effect analysis, we determined the numerical value of this inflection point. The risk of kidney stones in females increased with age up to 53 years (OR = 1.02, 95% CI 1.01–1.03), after which the risk decreased (OR = 0.98, 95% CI 0.97–0.99) (Table 4). Furthermore, inflection points were observed in curves for other milk consumption frequencies (“Never,” “Rarely,” “Sometimes”), occurring at similar time frames. Surprisingly, the curve for “Often” consumption frequency displayed a distinctly different pattern, consistently maintaining a lower prevalence level of kidney stones. Across all age groups of females, any milk consumption frequency, compared to the “never” curve, reduced the risk of kidney stone prevalence (Figure 3D). Additionally, we found that with the inflection point at 53 years as a boundary, more frequent milk consumption led to a significant decrease in kidney stone prevalence among females in both age segments (Figure 3E).

**Table 4:** Threshold effect analysis of age on kidney stones based on female using piecewise linear regression.

Inflection point of age, (years)	OR (95%CI)	*p*
<= 53	1.02 (1.01, 1.03)	<0.0001
> 53	0.98 (0.97, 0.99)	0.0006

OR, odds ratio; 95%CI, 95% confidence interval. Race, educational level, marital status, BMI, PIR, smoking, drinking, activity, hypertension, and diabetes were adjusted in model.

## 4 Discussion

To date, there have been no epidemiological studies reporting any association between milk consumption and kidney stone levels. In this cross-sectional study, which is nationally representative and based on the NHANES database, we observed that individuals who consumed milk more frequently had a reduced risk of developing kidney stones, with a more pronounced effect observed with higher degrees of milk skimming. Subgroup analyses revealed consistent results across all subgroups except for sex, where the protective effect of milk was evident only in women. The smooth fitting curve illustrated a nonlinear relationship between age and the prevalence of kidney stones among individuals with varying milk consumption levels. Notably, in women, the prevalence of kidney stones peaked at age 53 and then declined rapidly thereafter. Regardless of age, women who consumed milk exhibited lower rates of kidney stones compared to those who did not, particularly among regular milk consumers. These findings suggest that increasing milk intake may decrease the incidence of kidney stones, emphasizing the importance of milk consumption, especially among women.

Our study shows that milk is strongly associated with a reduced risk of kidney stones. It is well known that milk is a valuable source of many nutrients such as high-quality protein, calcium, vitamin D, magnesium, etc. (26). The most common stone phenotype is calcium-based, and the pathogenesis of kidney stones may be associated with metabolic pathways of calcium and vitamin D (13). However, studies on the relationship between the two and kidney stones are controversial (27). Previously, numerous prospective studies have indicated a strong correlation between increased dietary calcium intake and a decreased risk of kidney stones, a trend that is in line with our own findings (14–18). Among these studies, Borghi et al. observed that individuals with higher dietary calcium intake experienced a decrease in daily urinary oxalate excretion, thereby significantly lowering the likelihood of calcium oxalate stone formation (16). This phenomenon is attributed to the ability of dietary calcium to bind with dietary oxalates in the gastrointestinal tract, thereby reducing the absorption of oxalates and subsequent excretion in urine (28). Consequently, various organizations worldwide have consistently recommended that individuals with calcium stones ensure adequate dietary calcium intake, typically within the range of 1,000–1,200 mg daily (6). Moreover, balanced dietary calcium intake is advocated as a preventive measure against kidney stone formation (14). However, it is concerning that approximately 80% of the population does not meet the recommended dietary calcium intake established by the Institute of Medicine, which may further elevate the risk of developing kidney stones (18). Given that milk serves as a rich dietary source of calcium, our study suggests that maintaining a daily or more frequent milk consumption habit to some extent could help prevent the occurrence of kidney stones.

However, it is intriguing that Curhan et al. found that additional intake of calcium supplements could increase the risk of kidney stone formation (17). Randomized trial results have also indicated that daily supplementation with 1,000 mg of calcium carbonate and 400 IU of vitamin D may predispose individuals to kidney stones compared to a placebo (29). Notably, in this study, nearly two-thirds of participants took supplements between meals. Research has shown that when calcium supplements are taken with meals, they reduce the excretion of urinary oxalates (30). Hence, it can be speculated that the timing of calcium supplement intake, whether with meals or between meals, may have varying effects on the risk of kidney stone formation. Goldfarb suggests that the incidence of kidney stones may be lower if calcium supplements are administered in the form of citrate rather than carbonate. Regardless of whether the goal is to maintain bone density or prevent stone formation, dietary sources of calcium should be preferred over medications (31). The notable protective effect of dietary calcium against kidney stones may also be attributed to other dietary substances that could reduce the risk of stone formation. For instance, milk is rich in vitamin D, which enhances calcium absorption in the intestine. However, current prospective studies have indicated that vitamin D alone does not significantly affect the risk of kidney stones or hypercalcemia (32, 33). Indeed, considering that most individuals do not meet the recommended daily dietary calcium intake according to guideline recommendations (18), it may pose a challenge to discern the impact of vitamin D on promoting calcium absorption. Therefore, it’s essential to consider an individual’s calcium and vitamin D concentrations before opting for additional calcium intake and vitamin D supplementation.

Increasing fluid intake is a well-established and long-standing recommendation for the prevention of kidney stones (6). Research has indicated a correlation between the consumption of beverages such as coffee, tea, and alcohol in daily life and a reduced risk of kidney stone formation (34, 35). Furthermore, previous studies have suggested that milk consumption may alleviate the production of oxidative stress and inflammation (36, 37), both recognized as pivotal factors in the etiology of kidney stones (38). Studies have reported a close association between elevated blood lipids and an increased risk of kidney stone incidence (39), with our research demonstrating a more pronounced reduction in the risk of kidney stone occurrence correlating with higher levels of milk fat removal. Therefore, given milk’s acknowledged status as a healthful beverage, we strongly advocate for the consumption of at least one or more servings of low-fat milk daily to mitigate the occurrence of kidney stones.

In subgroup analyses, we observed sex differences (*P* for interaction = 0.033). Specifically, women may constitute the responsive group for increasing milk consumption to lower the risk of kidney stones (OR = 0.86, 95% CI 0.80–0.92), whereas men did not show a significant association (OR = 0.94, 95% CI 0.86–1.02). Presently, there is no evident explanation for this observation, suggesting the need for further investigation in future studies. Additionally, we identified a non-linear relationship between the prevalence of kidney stones and age in females using a smooth-fitting curve. Prior to menopause, the prevalence of kidney stones in women increased with age until menopause onset. This finding is consistent with conclusions from previous studies (40, 41) and may be linked to decreased estrogen levels in women (42). Research has indicated that women undergoing estrogen replacement therapy exhibit elevated urinary citrate levels and enhanced coagulation inhibition, potentially reducing the risk of kidney stone formation (43). However, postmenopausal women experience a gradual decrease in the prevalence of kidney stones with advancing age, a trend not reported in earlier studies (40–42). Surprisingly, postmenopausal women using hormone replacement therapy showed a higher tendency for kidney stone development compared to non-users, although statistical significance was lacking (40, 41). The current body of research cannot explain this observation, suggesting the necessity for further studies to elucidate the role of estrogen in postmenopausal women and associated mechanisms.

Furthermore, we observe an intriguing phenomenon in Figure 3D. Women who regularly consume milk exhibit a reversal of age-related changes in prevalence, maintaining a lower prevalence across all age ranges. We hypothesize that this observation may be linked to the magnesium content of milk. Hypomagnesuria is recognized as one of the known risk factors for kidney stone formation (6), and studies have demonstrated significantly lower 24-h urinary magnesium levels in postmenopausal women compared to premenopausal women (44). As the body ages, magnesium levels naturally decline. Magnesium deficiency has been associated with increased production of free radicals, consequently promoting oxidative stress and chronic inflammation (45), closely linked to the development of kidney stones (19). Adults are recommended to consume 320–420 mg of magnesium per day, and a single cup (234 ml) of low-fat milk contains approximately 33 mg of magnesium (46). Therefore, we underscore the importance of milk consumption, particularly advocating for the selection of low-fat milk, which may play a pivotal role in kidney stone prevention.

Our research presents several strengths. Firstly, it stands as the pioneering study to comprehensively evaluate the correlation between milk consumption and kidney stones. Secondly, our study incorporates extensive population data, considering complex sampling designs and weights to mitigate bias from oversampling, thereby ensuring the representativeness of the national population characteristics. Moreover, we adjust numerous covariates to enhance the accuracy of statistical analysis. Lastly, we conducted subgroup analyses to verify the stability of our results. Additionally, we plotted a smooth fitting curve, revealing the distinctive impact of milk consumption on kidney stone occurrence with advancing age. However, several limitations exist in this article. Firstly, the study’s cross-sectional design precludes establishing a cause-and-effect relationship. Secondly, reliance on self-reported data for both milk consumption and history of kidney stones introduces potential recall bias. Finally, there may be unobserved confounding factors, such as other types of dairy products or the influence of dietary calcium and other trace elements present in non-milk consumption sources. Due to limited data availability, these factors were not considered in our analysis. In future studies, it may be necessary to account for these potential confounders to ensure the rigor of the research conclusions.

## 5 Conclusion

Our research found that frequent milk consumption among women of all ages was linked to reduced rates of kidney stones. This discovery holds significant potential for public health interventions aimed at kidney stone prevention in women. However, due to the inherent limitations of cross-sectional studies, the robustness of this relationship is likely constrained and thus warrants validation through additional large prospective cohort studies.

## Data availability statement

The original contributions presented in the study are included in the article/supplementary material, further inquiries can be directed to the corresponding author/s.

## Ethics statement

All NHANES participants offered written informed consent, in alignment with the endorsement of the National Center for Health Statistics Research Ethics Review Board. The studies were conducted in accordance with the local legislation and institutional requirements. Written informed consent for participation in this study was provided by the participants’ legal guardians/next of kin.

## Author contributions

ZX: Writing – original draft, Writing – review & editing, Conceptualization, Data curation, Formal analysis, Software, Visualization. YZ: Conceptualization, Writing – original draft, Writing – review & editing. SP: Writing – original draft, Writing – review & editing. XZ: Writing – original draft, Writing – review & editing. GZ: Writing – original draft, Writing – review & editing. HJ: Writing – original draft, Writing – review & editing. CZ: Conceptualization, Funding acquisition, Supervision, Writing – original draft, Writing – review & editing. NC: Conceptualization, Funding acquisition, Supervision, Writing – original draft, Writing – review & editing.

## Footnotes

^1^ https://www.cdc.gov/nchs/nhanes

^2^ http://www.r-project.org/

^3^ http://www.empowerstats.com
